# Business angels and firm performance: First evidence from population data

**DOI:** 10.1371/journal.pone.0283690

**Published:** 2023-03-30

**Authors:** Magnus Lodefalk, Fredrik W. Andersson

**Affiliations:** 1 Örebro School of Business, Örebro University, Örebro, Örebro County, Sweden; 2 Ratio Institute, Stockholm, Stockholm County, Sweden; 3 Global Labor Organization, Essen, Düsseldorf, Germany; 4 Statistics Sweden, Örebro, Örebro County, Sweden; Babes-Bolyai University: Universitatea Babes-Bolyai, ROMANIA

## Abstract

Business angels dominate early-stage investment in firms, but research on their effects on firms is scarce and limited by sample selection. To address sample selection, we propose using population data and we develop an algorithm for identifying business angel investments in such data. We illustrate this novel approach by applying it to detailed and longitudinal total population data for individuals and firms in Sweden. In our application, we focus on a subset of business angels—active business angels who are themselves successful entrepreneurs with a profitable exit. We then study active business angels’ effects on firm performance, using population data. Employing a quasi-experimental estimator, we find that the business angels invest in firms that already perform above par. There is also a positive effect on subsequent growth compared with control firms. However, contrary to previous research on business angels, we cannot find any impact on firm survival. Overall, the paper underlines the need to address sample selection when studying business angels and suggests using population data for identification.

## Introduction

Business angels are instrumental for entrepreneurs in closing the gap between early-stage funding that entrepreneurs need and what is currently available [1, 2]. They account for the major part of early-stage investments in both Europe and the United States, with venture capital and crowd-funding investments accounting for the remainder [3, 4]. Despite their importance, rigorous research is scarce on the returns of business angels and the firms they invest in [4–6]. (For a literature review of business angel research, see, e.g., [6]). Overall, research on business angels is dominated by small-sample and often industry-specific studies, where angels typically are part of a business angel network. This introduces sample selection bias issues and affects the external validity of the research. The difficulty of identifying angels and the resulting reliance on small and non-representative samples is one of the reasons behind the retreat of business angel research [7].

In this paper, we propose to tackle the pertinent issue of sample selection in business angel research by exploiting the total population data of individuals and firms. We develop an algorithm to this end, and apply it to rich and longitudinal total population data for Sweden. In our application, we focus on a subset of business angels—entrepreneurs who turn into active business angels after a profitable exit. However, our algorithmic approach for population data could easily be broadened to include other types of business angels. We then meticulously employ a difference-in-difference matching estimator on data from Sweden to estimate the effect of business angel investment on firm performance. We analyse the effects on firm growth (jobs and sales), firm survival and the likelihood of becoming a high-growth firm (a so-called gazelle).

We contribute to the literature in three ways. To start with, we are first to set out to identify business angel investment using administrative population registers rather than surveys or information from business angel associations. Our algorithm may be applied and extended in other countries with micro-level data on individuals and firms. Such data, in the form of administrative register data from statistical agencies, are increasingly used in entrepreneurship and economics research [8, 9]. (For an overview of the potential role of such data in social sciences, see, e.g., [10]). Using an algorithmic and population-data based approach opens up a novel avenue for research on business angels. It also promotes replicable, data-driven and cost-effective research in the area. Second, we contribute by matching on a wider range of key firm characteristics than usual in the literature in our matching of firms that have received business angel investments (treated) with similar firms without such investments (controls). Notably, we match on firm growth trajectories, which we use as revealed measures for growth ambition. Finally, we contribute by studying the effect of angel investments not only on jobs and sales but also on firm survival as well as on the probability of becoming a gazelle.

Employing our difference-in-difference matching estimator, we find that firms with our type of business angel involvement show increased sales, employment and the likelihood of becoming a gazelle. Our finding of a pro-growth effect confirms two recent studies on business angels and refutes one that did not find a growth effect. However, contrary to recent studies, we do not find that our type of business angel investment affects firm survival. Overall, the paper points to the importance of considering sample selection in business angel research and suggests using population data for identification.

The remainder of the paper is organised as follows. In Section, we provide a primer on business angel investment and briefly review the literature on the effects of angels on firm performance. In Section, we present our population-based strategies for identifying business angels and their effects as well as descriptive statistics. In Section, we report and discuss the econometric results. In Section, we make concluding remarks.

## A primer and literature review

Business angels are private individuals who invest resources in new or smaller firms out of their own funds to yield a return [11]. To be called business angel investors, the investors are not to have any direct ties to the firms’ owners of the firms in which they invest [12–14]. Business angels may invest varying amounts of capital and other non-pecuniary resources. They can accordingly be classified as micro, knowledge-oriented, capital-oriented or classic business angels [15]. The four types differ in terms of the capital invested and the level of engagement in the firms [15]. A micro investor provides a minor amount of capital and takes an active, but minor, role in the firm. If the angel becomes more active, the business angel becomes a more knowledge-oriented angel. A capital-oriented business angel invests a larger sum of money but only takes a minor active role in the firm. If the business angel takes a more active stance, the investment becomes more similar to a classic business angel investment. As business angels mature, they can move on from being micro or knowledge-oriented investors to becoming more capital-oriented or classic business angel investors.

By investing their own funds in entrepreneurs’ endeavours, business angels reduce the equity funding gap. Business angels usually invest in firms that require more capital for future development than the owners can raise themselves. Compared to venture capital (VC) funds, business angels often invest a smaller amount than VC funds would [2]. As business angels invest their own capital, the principal-agent problem is attenuated, as there is no need to consider other stakeholders or investors, which can be a problem with VC funding [16]. Business angels are often believed to be more risk-averse than VC investors due to their limited financial assets, which reduces their ability to diversify risk [17]. Instead, their risk strategy is only to invest a minor part of their personal capital per investment, which delimits the risk of a potential negative outcome [18]. However, while business angels commonly invest relatively small parts of their capital, the risk associated with the investment may be substantial. Business angels fund early entrepreneurial and innovative activities with a high potential rate of return, and, by definition, such activities that can be expected to be relatively risky [19].

By investing non-pecuniary resources in entrepreneurial firms, business angels also contribute to the firms more generally. Business angels commonly take on an advisory or other responsible role, such as by joining the board of directors. In this way, business angels may share their experience, market knowledge and access to networks. Such involvement may generate additional advantages at a later stage, for example, by increasing the ability to attract capital from VC funds [20–22]. Though this may be a general pattern [22], highlight heterogeneity in the ability to attract VC funding, e.g., related the selectivity, monitoring and affiliations of business angels. Business angel involvement may also promote the survival of firms. Finally, in exchange for shares, business angel investment strengthens firms’ balance sheets [23].

Despite the fairly large literature on business angel investment, few studies rigorously analyse the effects of business angel investment [4, 6]. Instead, the focus is typically on returns to investors rather than to the firms they invest in. After reviewing the literature, [5] conclude that there is mixed evidence on business angels’ performance. For example [24], compared business angels’ investments with joint investments by business angels and VC funds in German-speaking countries and found that business angels did not receive higher returns compared with the joint investments.

Much of this literature on entrepreneurial funding suffers from methodological issues that severely limit the possibility of identifying causal relationships [6, 25, 26]. First, it is difficult to identify business angel involvement. Commonly, researchers turn to already available information from business angel networks [27]. However, this introduces a sample selection problem related to the business angels (only some business angels are selected) [28]. Second, it is a profound challenge to decide on the counterfactual case with which to compare the business angel investment. How do we assess whether a business angel promotes firm growth or merely picks the “winner”? This introduces sample selection issues in estimation of effects on firms (only the successful firms are selected). Below, we highlight four recent studies that contribute by paying attention to firm selection in terms of receiving business angel investment.

One study uses data from two business angel networks, consisting of approximately 370 US-based business angels in the years 2001–2006. A regression discontinuity approach is used to compare the year 2010 outcome for firms that barely received business angel funding with those that almost did (but ultimately did not) [26]. The authors find funded firms to be more likely to survive and to perform better in job growth, patenting and attracting website traffic. A subsequent study adopts a similar estimation approach using a data set for business angel investment in 21 countries. It finds business angel investment to be positively associated with job growth, the likelihood of firm survival and with firms subsequently obtaining additional funding [17].

Another study uses data from a French business angel network to study the effects of investments on 432 firms in the years 2008–2011, using a difference-in-difference estimator that compared random and matched (size, age, industry, region and capital structure) samples [5]. When comparing firms that received business angel investments with a random group of firms, firms with business angel investments exhibited significantly higher growth. However, in comparison with matched firms, there was no significantly higher growth for firms with business angel investment. In another paper that uses a difference-in-difference matching approach, the effects of business angels’ characteristics on 49 small Italian firms are studied, using 216 control firms that were matched on firm sales, sales growth, and year, country and industry indicators [29]. Data on business angels are from surveys performed by an business angel network in 2007 and data on firms from a commercial database for the period 2009–2016. The authors find that business angel entrepreneurial experience is positively associated with sales growth for high-growth but not slower-growth firms.

We conclude that recent studies considerably contribute to providing empirical, yet mixed, evidence on the effects of business angel investment. The key challenge in this literature is still the reliance on small-sample data, such as that from business angel networks and industries, and the resulting issue of sample selection bias. We address this issue by exploiting population data. Finally, for identification, we employ unusually detailed data to carefully implement a quasi-experimental estimator, and in doing so, also address the important issue of parallel trends.

## Identification and descriptive statistics

In this section, we propose exploiting administrative data from statistical agencies to identify business angel investment, using a four-step algorithm. We apply our algorithm to such data from Sweden. Next, we present our empirical framework for identifying the effects of such investment on firms. Finally, we provide descriptive statistics for business angels, the firms being invested in and control firms. (The statistical software package used in this paper is Stata, versions 16.0 and 16.1).

### Identification of business angel investment

To identify business angel investment and its effect requires longitudinal and population data to find the individuals with business experience who start to invest resources in a firm with which they are not previously connected, as well as data about those firms and similar control firms. Such data, in the form of administrative register data from statistical agencies, are increasingly used in entrepreneurship and economics research [8, 9]. (For an overview of the potential role of such data in social sciences, see, e.g., [10]).

We propose an algorithm that uses population data to match prospective business angels—individuals who both have business experience and capital—with prospective objects for investment, to arrive at business angels and firms with angel investment. The algorithm has four steps: (1) identify individuals with business experience; (2) ensure that they have resources to invest; (3) identify continuing firms that also have not experienced any major upheaval, e.g., mergers or acquisitions (this is to enable longitudinal analysis and to avoid contaminating any effects of business angels with, e.g., effects from an acquisition.) and (4) verify that the individuals remaining from (2) invest in a firm from (3). The algorithm necessitates relatively standard information on individuals (stock market portfolios, board memberships and general characteristics), firms (boards, board chairs, performances and general firm characteristics). It benefits from the presence of unique identifiers for individuals and firms. Additionally, it is advantageous with data from shareholder and multi-generation registers, if available.

To lay out the specifics of the approach and illustrate its usefulness, we apply the algorithm to administrative data from Statistics Sweden (SCB), the statistical agency of Sweden. We have ensured access to detailed longitudinal individual and firm-level data from Sweden for the period 2009 to 2015. The time period has been limited by our access to data on firms and individuals, with year 2015 being the latest available year. (We have performed robustness estimations over five different time periods (2015 − *t*, where *t* is between 1 and 5) to identify whether the entry of business angels, e.g., is associated with companies becoming fast-growing, so-called gazelle companies, while using a more parsimonious identification algorithm and estimation approach. Qualitatively, the results are robust, indicating a statistically significant and positive link between the entry of a business angel and the probability of becoming a gazelle firm).

The data encompass every individual in Sweden from 15 years of age and every limited company. Essentially, we match data from four administrative registers from Statistics Sweden. The matching does not rely on statistical matching methods, but instead benefits from the presence of the unique identifiers of individuals, plants, firms and enterprise groups in Sweden.

Shareholder and board member information comes from the Job Register of SCB. The Job Register is part of the Swedish Register-Based Labour Market Statistics of SCB that contains matched employer-employee data on all active firms and physical people in Sweden. We also use data from the Register of Executive Board Members of the Companies Registration Office. The Register of Executive Board Members is used to identify board members that are not captured in the Job Register as well as to identify new board members in prospective firms for business angel investment. From the Longitudinal Integrated Database for Health Insurance and Labour Market Studies we add data that, for example, enable us to ensure that the individual requirements are met. Finally we use the Dynamics of Firms and Workplaces Register of the SCB to ensure that the subsequent performance of firms with business angel investment can be observed, which requires that the firm continues to exist and has not been subject to mergers, acquisitions or splits.

Our specific application of the algorithm focuses on a subset of business angels, namely successful entrepreneurs who turn into active business angels after a profitable exit. This application ensures that those identified as business angels have recently experienced running a company and received a substantial increase in capital, as well as then actively engaged in another firm. The approach can easily be broadened to include other types of business angels. For example, the algorithm could be applied to population data to study angels that invest pre-existing wealth rather than profitable exit capital. Another extension would be to study passive business angels, conditional upon, shareholder register data for non-listed firms, data which unfortunately are lacking for Sweden.

In the following, we present the details of our four-step-algorithm by applying it to the previously described Swedish data. In the first and second step of the algorithm, we want to identify individuals with business experience and capital to invest. In the first step of our application, we therefore focus on shareholders and board members who have left a firm, thereby having entrepreneurial experience. We include individuals with experience from closely held firms as well as other firms. Starting with closely held firms, we identify individuals who previously were shareholders in a closely held limited company, based on Statistics Sweden’s Job Register. (The Job Register is the cornerstone of the register-based labour market statistics (RAMS). To be included as a shareholder in RAMS, the individuals must have received a salary from the limited company, and the company must have filed the required form (K10/KU31) to the Swedish Tax Authority). In this way, we identified 35,845 individuals who were shareholders in closely held limited companies in 2010, implying that they were also board members, but were no longer shareholders in 2012 or 2013. To ensure that the individuals left the companies, we added the restriction that they no longer were on the board of directors; this reduced the number of individuals to 28,248. To address under-coverage of the number of shareholders in closely held limited companies, we identified individuals who were board members in 2010 but ceased to be board members or chairs of limited companies by 2012, using the Executive Board Members Register and the Job Register. (Passive shareholders, who declare their capital gains or dividends on form K12, are never classified as shareholders in RAMS. This creates under-coverage of the number of shareholders of close limited companies in RAMS. In RAMS in 2015, 60 percent of limited companies were linked to at least one shareholder of a close limited company. To include board members from the remaining enterprises, we use the Companies Registration Office’s Register of Executive Board Members and the Job Register).

In this way, we identified 95,630 individuals who previously were on the board of directors of companies but were not any longer. (According to the Register of Executive Board Members, approximately 18 percent and 11 percent were also board chairs or managing directors, respectively). These 95,630 individuals were also not shareholders of limited companies in 2012, according to the Job Register. Summing up the first step, after removing duplicate individuals included both as previous shareholders and board members, we arrived at 117,221 unique individuals who ceased representing limited companies in the period 2010–2012.

In the second step, we identify individuals who not only have experience but also capital to invest. In our application, we require that the individuals who have ceased to represent limited companies also have left with substantial funds, enabling them to invest in other firms. We operationalise this by requiring that the individuals must have declared a capital gain of at least SEK 1.0 million (USD 102,753). Capital gains to be declared include, e.g., dividend payouts, stock sale gains, and interest income. (To avoid the capital windfall coming from a house sale, rather than a profitable firm exit, we require the individuals not to change their official postal address (2011–2012)). This reduced the number of individuals to 7,294. At this point, we would like to mention that we are somewhat conservative when defining business angels, aiming to fulfil the criterion of [15] that stipulates that business angels should invest in the new firms. However, an alternative and less stringent criterion could be based on the past performance of the individual’s previous firm, e.g., as indicated by that earlier firm having become a gazelle firm.

To recap, we have identified a group of individuals who previously were associated with a limited firm and who subsequently have had the potential to invest capital in other firms. We call these individuals prospective business angels. As mentioned, our application captures a subset of business angels. The application excludes individuals who invest in a new firm without recently having exited the board of another firm, or who exited without receiving a substantial increase in capital. In the presence of a shareholder register for non-listed firms, our first two steps could be broadened to also include such individuals. Additionally, in the presence of multi-generation register data, one could add the restriction that individuals must not be related to, e.g., management or board members of the firms in which they invest.

In the third step, we identify firms in which the prospective business angels may have invested. To capture the effects of business angels, we need the firms to exist as potential investment objects in the initial years and remain for the following years. Consequently, we added the restriction that the firms potentially invested in still had to exist in 2011–2012. We examined this aspect using data from the Dynamics of Firms and Workplaces Register (FAD). To separate the effects of business angels from other major changes in the firms, we also required that the firms did not replace their entire board of directors. Replacement of the entire board could indicate that the company has been acquired. We also formally required that the firms did not merge or split in the years 2011–2012, as indicated by the FAD register. Finally, we also required the limited companies to have at least one gainfully employed person, which is equal to being part of the firm population in RAMS.

In the fourth step, we want to identify actual new engagement in firms. In our application, we match prospective business angels that remain in step two with prospective investment objects from step three to identify active business angels. If the prospective business angel—an individual with experience and capital—also enters into the board of a prospective investment object—thereby actively engaging in the firm, we consider the individual to be a business angel for that firm. Using the Swedish Companies Registration Office’s Register of Executive Board Members, we searched for individuals who were appointed as new board members in the above-mentioned limited companies. We arrived at 297 prospective business angels who recently were appointed as board members of 357 prospective investment objects.

Applying our algorithm for total population data, we have identified novel and active engagement of carefully identified prospective business angels in likewise carefully identified prospective objects for investment. Henceforth, these individuals and firms are considered “business angels” and “firms with angel investment”. Having been appointed to the boards of directors, these business angels are expected to participate and assist in the firms’ strategic work. Presumably, the angels were approached by firms not only for pecuniary investment but also for their business knowledge, contacts, and corporate management experience. Such assets are likely valuable to these firms, for example, when working with their business plans. Accordingly, they meet a key criterion of [15] for knowledge-oriented business angels. Nevertheless, in the absence of a Swedish shareholder register for non-listed firms, our specific application cannot ascertain that the individuals actually used their recently received capital to invest in these firms. Therefore, our identified business angel investment in firms is an approximation for the subset of business angel investment that we have in mind, namely successful entrepreneurs that turn into active business angels after a profitable exit.

Finally, we make two adjustments to the data set. However, our results are robust without making these adjustments, with results available upon request. First, we are wary of capturing professional board member engagement—individuals engaged in a large number of firms—rather than typical business angels. Analysing the data, we find that our algorithm identifies a relatively large number of individuals engaging in firms in the industrial activities of head offices (NACE 7010), as well as in business and other management consultancies (NACE 7022). We recognise that these individuals are more likely to be professional board members than business angels. Therefore, we exclude these two 4-digit industries in the subsequent analysis. This reduces the number of individuals to 247 and leaves us with 300 unique firms. Second, we limited the analysis to SME firms, here defined as firms with less than 250 employees in 2011. To conclude, we, therefore, arrived at 156 firms and 134 individuals. Finally, to limit the influence of outliers on the results, we have excluded firms in the 1st and 99th percentiles of historical sales growth. We have also removed five observations with extreme growth in sales or employment from all estimations with a continuous response variable.

### Identification of effects on firm performance

We now turn to our strategy for identifying the effects of business angel investment on firm performance. As mentioned, a fundamental problem is potential selection bias from business angels “cherry-picking” their target firms. This is a problem in all evaluation studies of business angel investment, where firms do not simultaneously receive and not receive such investment. Ideally, we would like to obtain data on the unobserved outcomes, that is, on the counterfactual, to estimate unbiased causal effects from angel investment.

More formally, let *y*_*di*_ denote firm *i*’s outcome with treatment being indicated by the variable *d*_*i*_ = {0, 1}:

$$\begin{array}{c} y_{i}=y_{0i}+d_{i}\left(y_{1i}-y_{0i}\right) \end{array}$$ (1)

As we are interested in the effect of the treatment we can estimate the average treatment effect on the treated (*ATT*):

$$\begin{array}{c} {\hat{\delta }}_{ATT}=E\left(\left[y_{1i}-y_{0i}\right]|d_{i}=1\right)=E\left(y_{1i}|d_{i}=1\right)-E\left(y_{0i}|d_{i}=1\right) \end{array}$$ (2)

where *E*(⋅) denotes the mathematical expectation operator, that is, the population average of a random variable. In reality, we can only observe the first term on the right-hand side; that is, the average performance of firms with business angel investment of the type we are studying. The second term—the average performance of the counterfactual for non-treated firms, that is, the performance if they had received treatment—cannot be observed. However, we may still be able to construct a control group that enables us to provide a consistent estimate of the *ATT*. Put differently, we can estimate the change in the response variable as:

$$\begin{array}{c} \Delta =E\left(y_{1i}-y_{0i}|d_{i}=1\right)+\left[E\left(y_{0i}|d_{i}=1\right)-E\left(y_{0i}|d_{i}=0\right)\right] \end{array}$$ (3)

The expression is the sum of two components, the *ATT* plus a selection bias component. The selection bias component is included to account for the fact that the average firm performance of non-treated firms *E*(*y*_0*i*_∣*d*_*i*_ = 0) is not necessarily a good representation of the counterfactual case for firms that business angels chose to invest in *E*(*y*_0*i*_∣*d*_*i*_ = 1). The selection bias is zero if the outcomes firms from the treatment and comparison group would not differ in the absence of treatment. Therefore, *d*_*i*_ should ideally be randomly assigned among firms.

In the absence of randomisation, our strategy is to minimise selection bias by using a conditional difference-in-difference matching estimator (DD-PSM), that combines difference-in-differences (DD) with propensity score matching (PSM) [30–33]. (The approach is succinctly presented in [34]). In this way, we may draw an inference based on reconstructing the counterfactual, exploiting our rich observational data, to ensure conditional independence between the assignment of treatment and the control firms’ responses. A ‘*propensity score*’ is defined as the probability of a firm receiving treatment—business angel investment—and it is based on a vector of firm characteristics **x**, including the firm’s growth trajectories, and additionally controlling for industry-specific effects at the NACE-group level, i.e., slightly more granular than the two-digit industry level. (For the classification used, see Table A1 in S1 Appendix). We also impose a common support requirement, to ensure that firms with **x**-values have a positive and equal opportunity of being assigned to the treated and control groups [35]. We then estimate the *ATT* by first selecting two firms with the same propensity score *Pr*(*d*_*i*_ = 1∣**x**) = *p*(**x**), where one firm receives business angel investment, and the other does not, and then comparing the mean changes in performance for the treated and controls, that is:

$$\begin{array}{c} {\hat{\delta }}_{ATT}=E\left(y_{1i}|p\left(x\right)\right)-E\left(y_{0i}|p\left(x\right)\right) \end{array}$$ (4)

where the treatment effect on the treated is conditional on the propensity score.

In our vector of firm characteristics **x**, we include a range of important variables for business angel investment according to the literature [36, 37]. We control for the values of these variables in the year preceding business angel investment. The variables included are firm size (sales and employment), physical and human capital (tangible assets, and the wage bill for skilled workers as a proxy for research and development in the firm), operating returns (turnover ratio), solvency, leverage and whether the operating leader has a university degree and previous experience in that capacity. Most of these variables are related to the firm’s features while the last two focus on the skills and track record of the firm’s manager. (We also control for parallel trends in the matching procedure, by including growth in variables).

### Descriptive statistics

We now apply our algorithm for identifying business angel investment to study the association between our subset of business angels and firm performance in Sweden, using total population data. (Definitions and sources of our variables as well as their pair-wise correlations are provided in Tables A2 and A3 in S1 Appendix).

In Table 1, we present cross-sectional summary statistics for the two groups of control and treatment, before the nearest-neighbour matching takes place. As expected, before matching, the two groups of firms are rather similar but not identical. On average, the treated firms are more well-endowed in terms of size (sales and workforce), tangible assets, and human capital (education), while being more leveraged and displaying a lower turnover ratio. In terms of industries, the treated firms are more strongly represented in manufacturing, mining and quarrying, and in information and communication industries than control firms, see Table A4 in S1 Appendix. In addition, the control firms are more heavily represented in the construction and hospitality industries.

**Table 1:** Summary statistics for firms, 2011–2012.

	Control firms					Treated firms				
Variables	N	Mean	S.D.	Min	Max	N	Mean	S.D.	Min	Max
Ln Sales 2011	96,098	8.76	1.45	0.00	17.06	156	10.81	1.64	6.39	15.06
Ln Tangible assets 2011	96,098	5.89	2.73	0.00	17.17	156	8.70	2.70	0.00	17.28
Ln Wage highly edu 2011	96,098	5.59	6.45	0.00	18.63	156	12.50	5.29	0.00	18.22
Solvency ratio 2011	96,098	42.22	23.56	0.00	100.00	156	40.02	25.87	0.00	100.00
Turnover ratio 2011	96,096	2.36	2.62	-413.33	249.86	156	2.01	1.48	0.00	9.40
Leverage ratio 2011	96,098	3.51	9.02	0.00	100.00	156	7.26	17.95	0.00	100.00
Ln Workforce size 2011	96,098	1.57	1.18	0.00	5.52	156	3.14	1.28	0.00	5.51
Opf university degree	94,909	0.18	0.39	0.00	1.00	152	0.39	0.49	0.00	1.00
Opf experience of other Opf	94,909	0.18	0.39	0.00	1.00	152	0.38	0.49	0.00	1.00
Δ Sales	96,098	0.14	0.47	-8.98	4.86	156	0.31	0.51	-0.80	3.79
Δ Tangible assets	96,098	-0.03	1.35	-11.96	16.25	156	0.18	1.02	-3.36	4.97
Δ Wage highly edu	96,098	0.76	2.75	-7.03	16.40	156	1.03	3.18	-1.20	13.26
Δ Solvency ratio	96,098	0.78	17.39	-100.00	100.00	156	-3.18	22.81	-98.00	95.00
Δ Turnover ratio	96,096	-0.03	3.82	-918.65	244.58	156	0.11	0.77	-1.95	4.40
Δ Leverage ratio	96,098	-2.56	16.61	-100.00	100.00	156	-6.81	26.92	-99.80	80.70
Δ Workforce size	96,098	0.06	0.41	-4.06	4.54	156	0.15	0.38	-1.10	1.95

Table notes: The table presents cross-sectional statistics for the control and treatment firms, before matching takes place.

Before turning to the econometric results, we provide a snapshot of the firms being invested in and the angels investing. In Table 2, we note that the average firm is small-sized firms, and approximately a third of their workforce is female, and a quarter has post-secondary education. (Results from the propensity score matching are available in Tables A4-A6 in S1 Appendix. Based on the scores, we have chosen the nearest neighbours. Comfortingly, the bias in matching is low both overall and across variables, and it is never statistically significant at conventional levels).

**Table 2:** Characteristics of the firms being invested in.

Variable	Mean	S.D.	Min	Max
Employees	45.0	57.2	1	247
Post-secondary education	28.5	29.0	0	100
Female	32.5	24.5	0	100

Table notes: The table presents statistics for the firms being invested in by business angels, with variables in percent, except for workforce size, which is in number of employees.

Turning to the angels, in Table 3, an overwhelming majority of them are male and middle-aged. Most of them hold a post-secondary degree, commonly in the social sciences.

**Table 3:** Characteristics of the business angels.

Variable	Mean	S.D.	Min	Max
Female	3.6	18.8	0	1
Age	54.3	9.2	34	74
Post-secondary education	65.7	47.6	0	1
Social sciences degree	48.9	50.2	0	1
Natural sciences/Engineering degree	35.8	48.1	0	1
Health and welfare degree	2.9	16.9	0	1

Table notes: The table present statistics on the business angels, with variables in percent, except for age, which is in years.

## Econometric results

Our estimates of the effects of business angel investment on firms are presented in Table 4, with the panels corresponding to the four response variables. For each variable, we present results from: a simple test for any difference between the two groups before firm-to-firm matching; an OLS regression, also before such matching; and then from employing four DD matching estimators. Our preferred estimator is the first nearest-neighbour estimator, which aims to minimise bias.

**Table 4:** Effects of business angel investment on firm performance.

	Treated (1)	Controls (2)	ATT (3)	S.E. (4)	*t*-stat (5)	Obs. (6)
(A) *Change in employment*						
All firms	14.21	4.38	9.83	3.68	2.67	96,254
OLS			10.87	4.33	2.51	95,059
First Nearest Neighbour	14.27	0.73	13.22	5.09	2.59	152
Four Nearest Neighbour	14.27	5.96	8.31	4.45	1.87	152
Weighted Nearest Neighbour			12.30	5.86	2.10	152
Kernel matching			10.27	4.48	2.29	152
(B) *Change in sales*						
All firms	18.76	6.58	12.18	3.45	3.53	96,254
OLS			8.59	3.84	2.24	95,059
First Nearest Neighbour	18.44	7.76	9.96	4.41	2.26	152
Four Nearest Neighbour	18.44	9.99	8.45	3.89	2.17	152
Weighted Nearest Neighbour			12.70	5.18	2.45	152
Kernel matching			8.66	3.98	2.18	152
(C) *(0,1) Gazell*						
All firms	0.08	0.02	0.06	0.01	5.98	96,254
Logit (odds ratio)			2.13	0.68	2.38	95,024
First Nearest Neighbour	0.09	0.02	0.07	0.02	2.77	152
Four Nearest Neighbour	0.09	0.03	0.05	0.02	2.38	152
Weighted Nearest Neighbour			0.06	0.03	2.22	152
Kernel matching			0.05	0.02	2.46	152
(D) *(0,1) Survival*						
All firms	0.84	0.83	0.01	0.03	0.41	121,560
Logit (odds ratio)			0.94	0.56	-0.10	105,771
First Nearest Neighbour	0.98	0.97	0.01	0.02	0.71	166
Four Nearest Neighbour	0.98	0.98	0.00	0.01	0.38	166
Weighted Nearest Neighbour			0.06	0.02	2.55	166
Kernel matching			0.00	0.01	0.11	166

Table notes: The table presents a simple comparison between treated and control firms and then average treatment effects on the treated. We use five estimators, i.e., an OLS estimator and four DD matching estimators, the latter with replacement (except when four nearest-neighbour matches are used). The response is measured as the difference in outcomes between 2012 and 2015. The estimator’s control for industry-specific effects. A common support restriction has also been imposed. Robust standard errors are used when employing OLS and the first two DD matching estimators, while bootstrapped standard errors (with 500 replications) are used when employing the last two estimators. The response variable is binary in panels (C)-(D).

In Panels (A) and (B), we analyse the employment and sales growth of firms that receive business angel investment (treated) and those that do not (controls). We find that treated firms experience substantially higher employment and sales growth than do control firms. Overall, the results are statistically significant at conventional levels and across estimators. Firms with business angel investment grow almost 10–13 percent more than similar firms without such investment.

Next, in Panel (C), we analyse if firms’ stronger growth performance with angel investment also translates into them having a higher likelihood of becoming high-growth firms (so-called gazelles). In recent years, gazelle firms have both received a great deal of attention from policy-makers and in research. The presence of gazelles has been associated with substantial job creation [38]. Gazelle firms are in the OECD statistics defined as firms annually growing by 20 percent over three years, following [39]. We operationalise the concept by applying the OECD definition of employment, while using an alternative definition for the micro-sized firms’ subset (growing with seven or more employees), to heed the issues and recommendation of [40]). We find that few firms are likely to become gazelles, irrespective of any business angel investment. However, the likelihood of becoming a gazelle is higher for firms with business angel investment. Moreover, the difference is non-trivial and statistically significant across estimators.

Finally, in Panel (D), we focus on whether business angel investment helps firms survive. Descriptively, we have noted that high-growth firms are associated with somewhat lower survival rates, suggesting ambition coming together with a higher risk of failure. It would, therefore, be advantageous for ambitious firms if business angel engagement would assist them in surviving. According to previous studies on samples from business angel networks, business angel investment does attenuate the risk of failure. We, revisit this issue, using population data. In line with the previous studies, we find that business angel investment is associated with a higher probability of survival. However, contrary to those studies, the association to survival is economically trivial and statistically insignificant. Therefore, we conclude that we cannot establish any particular association between the investment by business angels and the survival rates of the firms they invest in, compared with similar firms without such investment.

We conclude that the presented results confirm those of two recent studies, which suggest that business angel investment spurs growth, while refuting the results of another study, which does not find such a pattern [5, 17, 26]. In contrast to the recent studies, we cannot find any impact on subsequent firm survival, whether positive or negative. We argue that such an impact is not necessarily expected since business angel investment could be associated with both ambitions and riskier behaviour. An absence of such an effect in tandem with a pro-growth effect would suggest that business angel investment is advantageous for firms subject to it—promoting growth while simultaneously avoiding to raise the risk of firm exit.

## Concluding remarks

Business angels have an instrumental role in reducing the equity funding gap for smaller enterprises. Business angels may become especially important in the aftermath of the COVID-19 crisis. In the crisis, financing has been squeezed, and bankruptcies, as well as layoffs, have multiplied, and this could stimulate a post-crisis increase in entrepreneurship as well as an increased demand for the funding of entrepreneurial activities. Generally, the funding of small and medium enterprises (SMEs) is important, since SMEs account for the majority of firms, employ a majority of workers and importantly contribute to innovation and job creation [41].

Previous research on the effects of business angel investment has faced difficulties in providing representative findings due to the lack of population-based data on business angels. Relying on data samples from business angel networks and specific industries, the existing evidence for a positive effect of business angels on firm performance is mixed. The issues with research in this area have received attention from international organisations. The [42] writes that *“much of the available evidence on angel activities is anecdotal. It often comes from surveys and may be inaccurate in part due to limited or unrepresentative samples of the overall angel population* [43], *which also might explain why angel activities sometimes receive less attention from researchers and policy makers than other sources of finance.”* Thus, strengthening research on business angel investment is important also for stimulating policy-makers’ interest, and ultimately for countries promoting entrepreneurial business’ access to funding for firm growth.

This paper proposes exploiting administrative and population-based registers to identify business angels and their effects on firm performance. We present an algorithm to this end. We illustrate its usefulness by applying it to study a subset of business angels—successful entrepreneurs with a profitable exit that actively engage in a new firm. We then estimate the effects of these business angels on firm performance, carefully using a difference-in-difference matching estimator. Our results confirm a pro-growth effect on firms but cannot confirm any substantial effect on firm survival.

Beyond applying the proposed approach of using population-based data in business angel research to other countries, the approach could be used to investigate additional implications of business angel investment. For example, having identified business angels without the limit of sample selection, research on the implications for firm dynamics, human capital accumulation as well as employee remuneration would seem worthwhile. Moreover, the proposed approach could be improved by exploiting multi-generation registers to ensure that family ties are controlled for. Finally, the proposed approach could be extended in a number of ways to include other types of business angel investors and to study their potentially heterogeneous effects on firms.

There are three main limitations to our study, which relate to the specific application on Swedish data. In the presence of additional data, researchers could easily address these issues when adopting the proposed population-based approach to business angel research. Starting with the first limitation of our application, we highlight that in the absence of a Swedish shareholder register for non-listed firms, we have only been able to study a subset of business angels—active business angels who are themselves successful entrepreneurs with a profitable exit. Our econometric analysis therefore excludes individuals who exited a firm without receiving a substantial increase in capital. Second, the absence of a non-listed firm shareholder register has also disabled us from establishing that the individual who is observed to invest non-pecuniary resources in another firm also invests his or her recently received pecuniary capital in the same firm. Third and finally, without access to a multi-generation register, we have not been able to remove individuals that are related, e.g., to the management of the firms in which they invest. The second and third limitations have potentially made us slightly overestimate the number of Swedish business angels, and slightly underestimate the impacts of business angels on firm performance. (Including individuals who have not necessarily invested pecuniary capital likely biases the results downwards, as does the inclusion of individuals who may be related, and hence bringing in less “foreign” non-pecuniary capital to the investee than other business angels).

To conclude, this paper lays out a novel way of addressing sample selection when studying business angels and their investment subjects. It illustrates the approach by applying it to comprehensive Swedish population data, enabling the study of a subset of business angels and their impacts on investees. We hope the paper will stimulate population-based research to improve the identification of business angels and their effects on firm performance. Solid research evidence is important to increase policymakers’ interest in the funding of entrepreneurial activities. Having improved the identification of business angels, it is easier to formally evaluate public policies and activities to promote business angel investment, establishing which policies work and which do not. Combining solid research and evaluations will arguably be instrumental for devising policies to reduce the equity funding gap, to the benefit of both firms and employees. An active business angel market can be of particular importance in the COVID-19 post-crisis time.

## Supporting information

S1 Appendix Descriptive information and statistics. (PDF)
