# Supplementary material for: Business angels and firm performance: First evidence from population data
Source: PLoS One. 2023 Mar 30;18(3):e0283690. doi: 10.1371/journal.pone.0283690 (PMC10062610; doi:10.1371/journal.pone.0283690)
Supplement: S1 Appendix — (PDF) [file pone.0283690.s001.pdf]

S1 Appendix

Business Angels and Firm Performance: First Evidence from Population Data

Fredrik W. Andersson

Magnus Lodefalk

January 27, 2023

S1 Appendix: Descriptive information and statistics

Table A1
Industrial definitions

NACE-codes	Industry description
01	Ariculture, forestry and fishing
02	Mining and quarrying
02	Manufacturing
03	Electricity, gas, steam and air conditioning supply
04	Water supply; sewerage, waste management and remediation activities
04	Construction
05	Wholesale and retail trade; repair of motor vehicles and motorcycles
06	Transportation and storage
07	Accommodation and food service activities
08	Information and communication
09	Financial and insurance activities
10	Real estate activities
11	Professional, scientific and technical activities
11	Administrative and support service activities
12	Public administration and defence; compulsory social security
13	Education
14	Human health and social work activities
15	Arts, entertainment and recreation
15	Other service activities
15	Activities of households as employers; undifferentiated goods- and services-producing activities of households for own use
15	Activities of extraterritorial organisations and bodies

Notes: The table presents our industrial aggregation and is based on the NACE (Rev.2), which is the statistical classification of economic activities in the EU.

Table A2
Variable definitions and data sources

Control variables	*Definitions*	*Sources*
Ln Sales 2011	Ln (firms' net sales, 2011)	SBS
Ln Tangible assets 2011	Ln (firm's tangible assets 2011)	SBS
Ln Wage highly edu 2011	Ln (firm's wage bill for workers with post-secondary education)	RAMS
Solvency ratio 2011	Shareholders' equity over total assets, in percent, 2011	SBS
Turnover ratio 2011	Net turnover over total assets, in percent, 2011	SBS
Leverage ratio 2011	Total debt over total assets, in percent, 2011	SBS
Ln Workforce size 2011	Ln (firm's number of employees)	RAMS
Opf university degree (0,1)	Operating leader (OPF) has university degree (1,0), 2011	RAMS
Opf experience (0,1)	OPF has been OPF in another firm (1,0) in 2009-2011	RAMS
Δ Sales	Ln Sales 2011 - Ln Sales 2009	SBS
Δ Tangible assets	Ln Tangible assets 2011 - Ln Tangible assets 2009	SBS
Δ Wage highly edu	Ln Wage bill highly edu 2011 - Ln Wage bill highly edu 2009	RAMS
Δ Solvency ratio	Solvency ratio 2011 - Solvency 2009	SBS
Δ Turnover ratio	Sales over total assets 2011 - Sales over total assets 2009	SBS
Δ Leverage ratio	Leverage ratio 2011 - Leverage ratio 2009	SBS
Δ Workforce size	Ln Workforce size 2011 - Ln Workforce size 2009	RAMS
Outcome variables	*Definitions*	*Sources*
Change in employment	Change in employment, 2012-2015	SBS
Change in sales	Change in sales, 2012-2015	SBS
Gazelle (0,1)	Annual employment growth $\geq$ 20 percent, over the 2012-2015 period; except for micro-firms, see text	SBS
Survival (0,1)	Firm remaining in the FDB, in year 2015	FDB

Notes: The table presents variable definitions and sources. The sources from Statistics Sweden are Structural Business Statistics, SBS; Register-based Labour Market Statistics, RAMS; and Business Register, FDB.

Table A3
Pairwise correlations

Variable	No.	1	2	3	4	5	6	7	8	9	10	11	12	13	14	15	16
Ln Sales 2011	1	1.000															
Δ Sales	2	0.189	1.000														
Ln Tangible assets 2011	3	0.511	0.034	1.000													
Δ Tangible assets	4	0.068	0.150	0.362	1.000												
Ln Wage highly edu 2011	5	0.369	0.058	0.169	0.037	1.000											
Δ wage highly edu	6	0.066	0.116	0.028	0.039	0.258	1.000										
Solvency ratio 2011	7	-0.203	-0.106	-0.094	-0.024	-0.027	-0.049	1.000									
Δ Solvency ratio	8	-0.047	-0.057	-0.049	-0.077	-0.024	-0.008	0.296	1.000								
Turnover ratio 2011	9	0.122	0.068	-0.214	-0.075	0.001	0.030	-0.187	-0.001	1.000							
Δ turnover ratio	10	0.014	0.108	0.001	-0.047	0.005	0.006	-0.006	0.008	0.401	1.000						
Leverage ratio 2011	11	0.106	0.015	0.098	0.016	0.060	0.017	-0.359	-0.126	0.010	-0.002	1.000					
Δ leverage ratio	12	-0.05	-0.029	-0.026	0.013	-0.038	0.005	-0.001	0.343	-0.007	-0.013	0.217	1.000				
Ln Work force size 2011	13	0.839	0.132	0.467	0.061	0.413	0.090	-0.231	-0.061	0.104	0.009	0.107	-0.039	1.000			
Δ work force size	14	0.103	0.413	0.042	0.129	0.072	0.163	-0.077	-0.06	0.027	0.024	0.024	-0.006	0.229	1.000		
Opf university degree	15	0.035	0.005	0.006	0.014	0.542	-0.048	0.063	-0.002	-0.065	0.000	0.027	-0.017	0.029	0.002	1.000	
Opf experience of other Opf	16	0.174	0.030	0.146	0.023	0.114	0.045	-0.116	-0.017	-0.017	0.005	0.097	-0.02	0.167	0.032	0.060	1.000

Notes: The table displays pair-wise correlations between key variables.

Table A4
The distribution of firms across industries

	Control firms		Treated firms	
Industry	No.	Percent	No.	Percent
01	3,166	3.29	1	0.64
02	12,364	12.87	33	21.15
03	581	0.60	4	2.56
04	15,431	16.06	8	5.13
05	22,525	23.44	30	19.23
06	6,761	7.04	8	5.13
07	4,067	4.23	2	1.28
08	5,33	5.55	19	12.18
09	33	0.03	2	1.28
10	2,965	3.09	6	3.85
11	14,708	15.31	22	14.10
12	0	0	0	0
13	1,505	1.57	3	1.92
14	3,468	3.61	7	4.49
15	3,194	3.32	11	7.05

Notes: The table presents the distribution of control and treatment firms across the industries of table A1., aggregated to the two-digit level.

Table A5

Logit estimates and mean characteristics for the growth models

	Logit coefficient (1)	S.E. (2)	Treated firms (3)	Control firms (4)	Percent bias (5)	t-stat (6)	$p > \|t\|$ (7)
Ln Sales 2011	**0.440**	0.111	10.78	10.67	7.20	0.59	0.55
Ln Tangible assets 2011	0.118	0.053	8.66	8.59	2.70	0.24	0.81
Ln Wage highly edu 2011	**0.081**	0.023	12.44	12.68	-4.10	-0.43	0.67
Solvency ratio 2011	0.002	0.004	40.01	40.66	-2.60	-0.23	0.82
Turnover ratio 2011	-0.117	0.071	2.03	2.08	-2.50	-0.30	0.76
Leverage ratio 2011	0.002	0.006	7.27	7.52	-1.70	-0.12	0.91
Ln Workforce size 2011	0.063	0.122	3.12	3.07	3.40	0.28	0.78
Opf university degree	-0.054	0.192	0.39	0.39	-1.50	-0.12	0.91
Opf experience	0.345	0.173	0.38	0.36	6.00	0.47	0.64
Δ Sales	0.311	0.191	0.29	0.26	6.10	0.48	0.63
Δ Tangible assets	-0.011	0.077	0.17	0.13	3.40	0.35	0.73
Δ Wage highly edu	0.006	0.03	0.97	1.21	-8.40	-0.66	0.51
Δ Solvency ratio	-0.003	0.005	-3.34	-3.72	1.90	0.14	0.89
Δ Turnover ratio	0.105	0.072	0.11	0.12	-0.10	-0.02	0.99
Δ Leverage ratio	0.00	0.004	-6.40	-5.50	-4.10	-0.29	0.77
Δ Workforce size	-0.029	0.237	0.14	0.16	-5.40	-0.49	0.62
Observations	94,989		152	152			

Notes: The table displays estimates of the propensity to receive business angel investment for the sales and employment growth DD matching estimations. We use logit estimation and condition on the pretreatment characteristics. Our DD matching estimator employs one nearest-neighbour matching without replacement. A common support restriction is also imposed. We control for industry specific effects. The average mean bias and median bias per variable is 4.7 percent and 4.0 percent, , respectively. Pseudo-R^2 is 0.1615. For coefficients in bold, $p < 0.01$.

Table A6

Logit estimates and mean characteristics for the firm survival models

	Logit coefficient (1)	S.E. (2)	Treated firms (3)	Control firms (4)	Percent bias (5)	t-stat (6)	$p > \|t\|$ (7)
Ln Sales 2011	**0.490**	0.103	10.82	10.84	-1.30	-0.11	0.91
Ln Tangible assets 2011	0.092	0.049	8.62	8.36	9.30	0.84	0.40
Ln Wage highly edu 2011	**0.082**	0.022	12.51	12.79	-4.90	-0.54	0.59
Solvency ratio 2011	0.001	0.004	39.78	39.83	-0.20	-0.02	0.98
Turnover ratio 2011	-0.099	0.066	2.08	2.47	-19.10	-1.73	0.08
Leverage ratio 2011	0.001	0.005	6.95	7.74	-5.70	-0.39	0.70
Ln Workforce size 2011	0.041	0.113	3.10	3.14	-2.80	-0.24	0.81
Opf university degree	0.066	0.182	0.41	0.33	17.60	1.48	0.14
Opf experience	0.326	0.166	0.38	0.45	-15.00	-1.23	0.22
Δ Sales	0.219	0.183	0.28	0.26	3.50	0.29	0.77
Δ Tangible assets	0.019	0.071	0.19	0.22	-3,00	-0.28	0.78
Δ Wage highly edu	0.019	0.027	1.06	1.54	-16.00	-1.25	0.21
Δ Solvency ratio	0.000	0.000	-2.89	-2.37	-2.50	-0.22	0.82
Δ Turnover ratio	0.088	0.067	0.10	0.08	1.40	0.23	0.82
Δ Leverage ratio	-0.002	0.004	-6.72	-5.21	-6.90	-0.55	0.58
Δ Workforce size	-0.013	0.225	0.13	0.12	3.70	0.34	0.74
Observations	105,692		166	166			

Notes: The table displays estimates of the propensity to receive business angel investment for the firm survival DD matching estimations. We use logit estimation and condition on the pretreatment characteristics. Our DD matching estimator employs four nearest-neighbour matching without replacement. We control for industry specific effects. A common support restriction is also imposed. We control for industry specific effects. The average mean bias and median bias per variable is 5.8 percent and 3.5 percent, , respectively. Pseudo-R^2 is 0.1618. For coefficients in bold, $p < 0.01$.
